# ﻿A new freshwater amphipod (Arthropoda, Malacostraca, Amphipoda, Gammaridae), *Echinogammarusozbeki* sp. nov. from the Tomara Waterfall, Turkey

**DOI:** 10.3897/zookeys.1173.102946

**Published:** 2023-08-04

**Authors:** Hazel Baytaşoğlu

**Affiliations:** 1 Recep Tayyip Erdogan University, Faculty of Fisheries and Aquatic Sciences, 53100 Rize, Turkiye Recep Tayyip Erdogan University Rize Turkiye

**Keywords:** Freshwater, morphology, new taxa, taxonomy

## Abstract

A new species of freshwater amphipod, *Echinogammarusozbeki***sp. nov.**, collected from Tomara Waterfall, Gümüşhane province, northeastern Anatolia, is described and illustrated. The new species belongs to the genus *Echinogammarus* and exhibits characteristic uropod 3 features of that genus. Some of the distinctive features of the *Echinogammarusozbeki***sp. nov.** species are a small body length, urosome segments without dorsal elevations, distal end of the peduncle segments of antenna 1 with setae longer than the diameter of the segment, presence of strong spines accompanying setae on both inner and outer margin of exopod, and uropod 3 parviramous.

## ﻿Introduction

A waterfall is a landform that occurs when water falls down a long or short distance due to the slope of the riverbed. Waterfalls, which are natural wonders, not only possess tourist potential but also serve as subjects for scientific studies in terms of geology, geography, phytology, and zoology. While Türkiye is one of the countries with high potential in terms of natural beauty and biodiversity, there are still deficiencies regarding the biodiversity of water resources (Doğanay 2011). Taxonomy plays a crucial role in determining biological diversity and consequently in the development of conservation plans. The topographic and climatic diversity that Türkiye possesses has led to a high species diversity, providing opportunities for the differentiation of species, especially those with limited mobility (Tavşanoğlu 2016).

Species of the order Amphipoda belonging to marine, freshwater, estuarine, cave, and underground water sources are frequently encountered. Amphipoda are represented by 16 families and 31 genera, and Gammaridae is the most commonly encountered family with 64 species in water resources in Türkiye (İpek and Özbek 2022). *Echinogammarus* is a genus of Gammaridae with high endemism, mostly inhabiting freshwater and brackish waters; only a few species are marine. The genus *Echinogammarus* comprises 58 taxa belonging to three main groups: the *berilloni* group, the *simoni* group, and the *pungens* group. Until now, species records belonging to this genus have been recorded from many regions, such as Europe, Algeria, Türkiye, the Iberian Peninsula, and North Africa (Pinkster 1993). According to recent studies, there are seven species belonging to the genus *Echinogammarus* in Türkiye: *E.antalyae* G. Karaman, 1971, *E.baliki* Özbek & Ustaoğlu, 2007, *E.foxi* (Schellenberg, 1928), *E.ischnus* Stebbing, 1899, *E.trichiatus* (Martynov, 1932), *E.stocki* G. Karaman, 1970, and *E.veneris* (Heller, 1865). Two of these species (*E.antalyae* and *E.baliki*) are endemic (İpek and Özbek 2022).

This study examines the individuals of *Echinogammarus* collected from Tomara Waterfall, Gümüşhane province, in terms of morphological features. Detailed descriptions and drawings are given of the extremities of the holotype male of a newly identified species, and the morphology of this new species is compared with its relatives.

## ﻿Materials and methods

**Figure 1.**
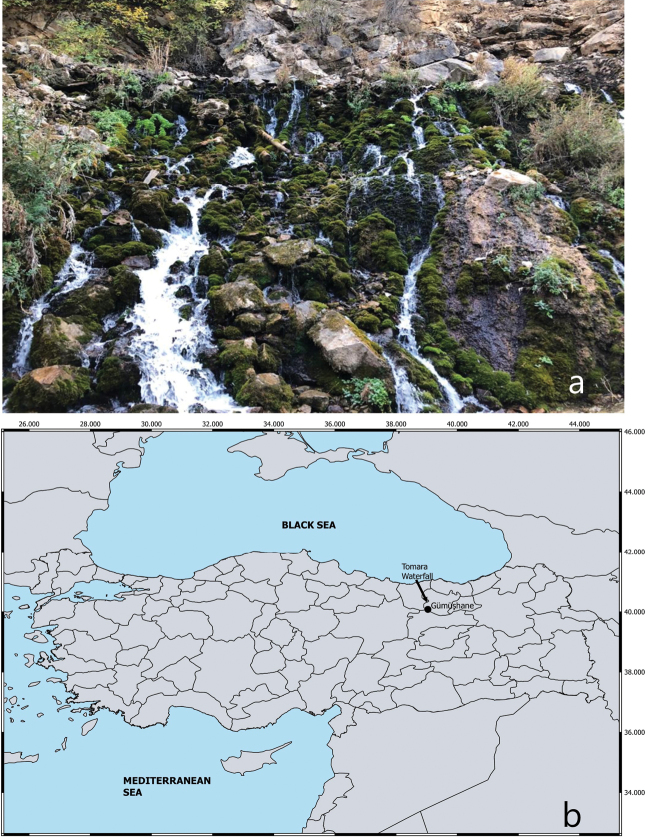
**a** habitat at the type locality of *Echinogammarusozbeki* sp. nov. **b** location of Tomara Waterfall in Türkiye.

Located within the borders of Şiran district of Gümüşhane province, Tomara Waterfall has an area of 70,000 m^2^ and flows from an altitude of approximately 1380 m. Its source is underground waters which pass through a deep, narrow valley and flow into Kelkit stream. The habitat is rocky, and vegetation is quite abundant. (Fig. 1a).

The distance between Gümüşhane and Şiran is 101 km, and the distance between Şiran and Tomara Waterfall is 21 km. There is an area available for visitors along the river that continues from the waterfall. To reach the Tomara Waterfall on foot from the lower basin of the river, the distance I approximately 1 km. The waters of this waterfall emerge from a karstic source, and its geological structure consists of layered sedimentary schists, limestone, sandstone, and marl (Doğanay 2011).

Specimens of *Echinogammarus* were collected with a fine-mesh hand net, fixed in 4% formaldehyde in the field, and later examined and dissected under a stereomicroscope. The body length is measured from the basis of antenna 1 to the basis of the telson, keeping holotype male straight (Hou et al. 2005). Morphological photographs were taken with a digital camera attached to a microscope. The drawings were made with the aid of a drawing tube mounted on a compound microscope. The type specimens have been deposited in the Recep Tayyip Erdoğan University Fisheries Museum.

## ﻿Results

### ﻿ Echinogammarus ozbeki sp. nov.

Taxon classificationAnimaliaAmphipodaGammaridae

734867C3-FD03-5509-B9BD-89E44DFE8D85

https://zoobank.org/5585E361-E857-4808-ABDB-FC7143F93AAB

**Figure 2.**
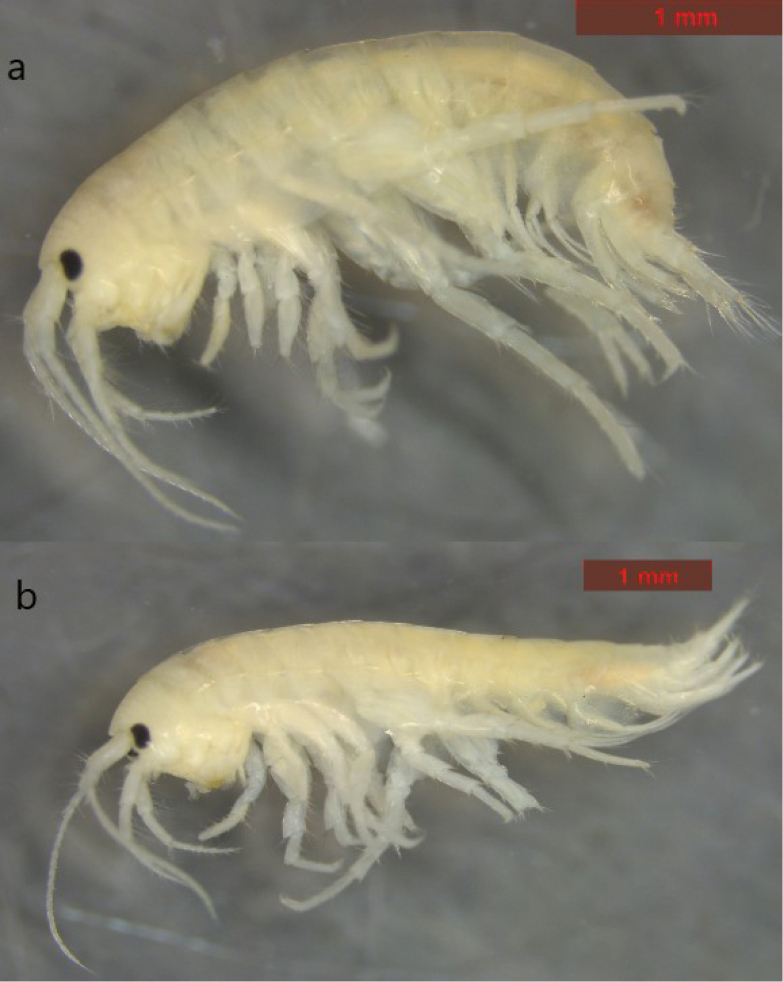
*Echinogammarusozbeki* sp. nov. **a** male **b** female.

**Figure 3.**
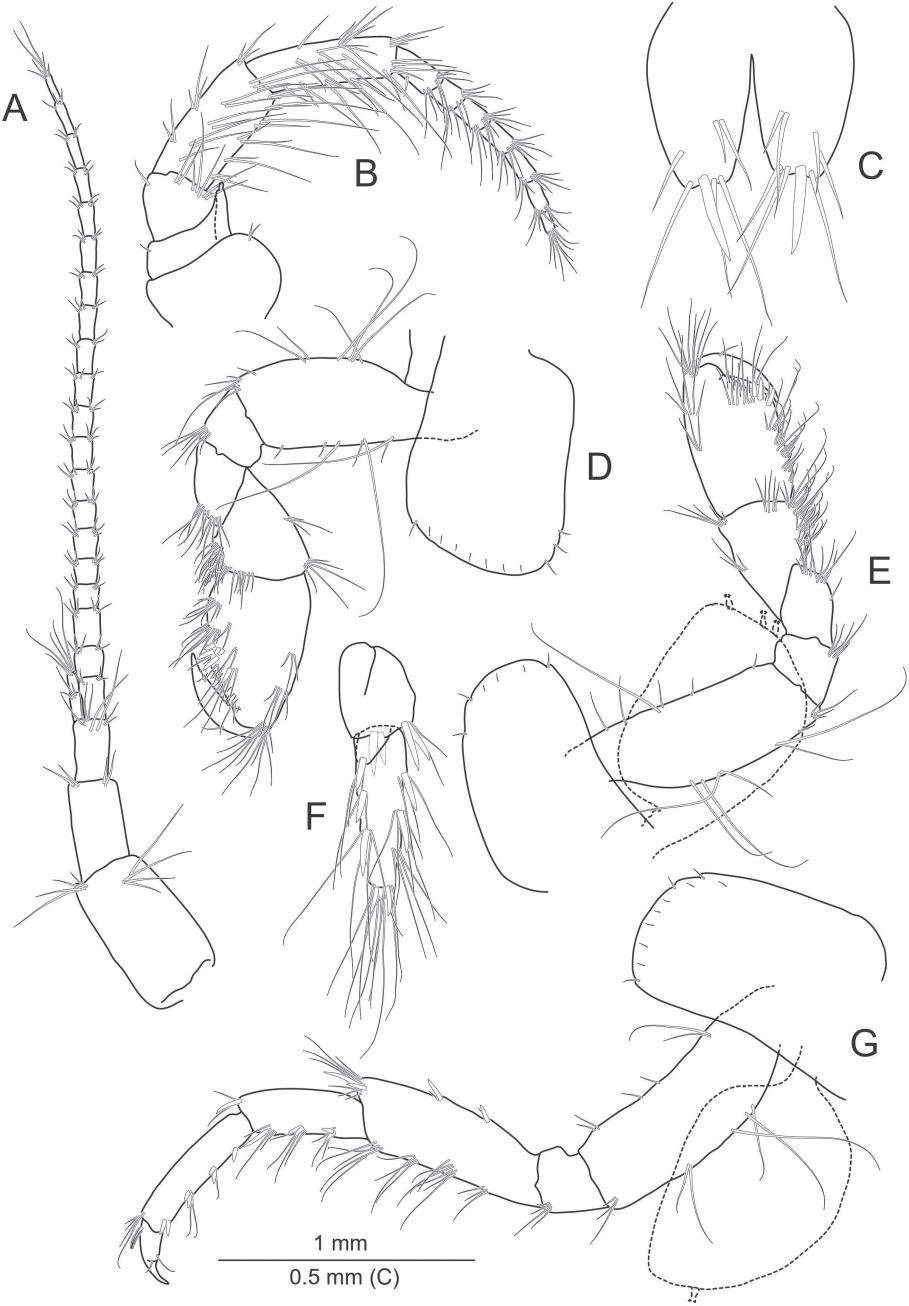
*Echinogammarusozbeki* sp. nov. holotype male **A** antenna 1 **B** antenna 2 **C** telson **D** gnathopod 1 **E** gnathopod 2 **F** uropod 3 **G** pereopod 3.

**Figure 4.**
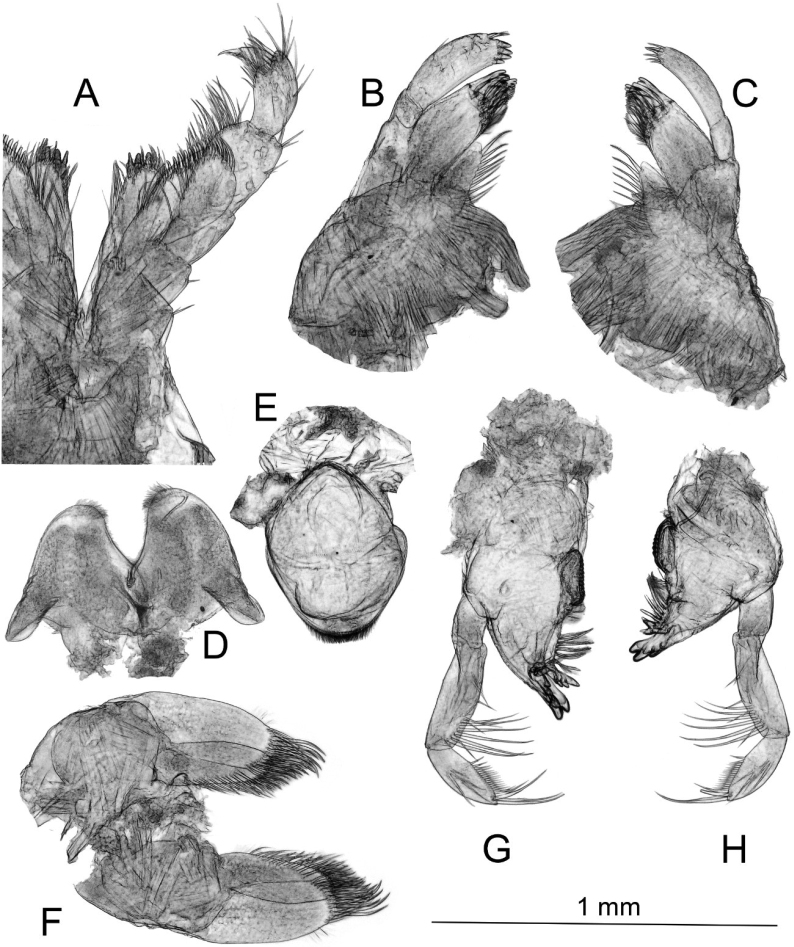
*Echinogammarusozbeki* sp. nov. holotype male **A** maxilliped **B** right maxilla **C** left maxilla **D** lower lip **E** upper lip **F** maxilla 2 **G** right mandible **H** left mandible.

**Figure 5.**
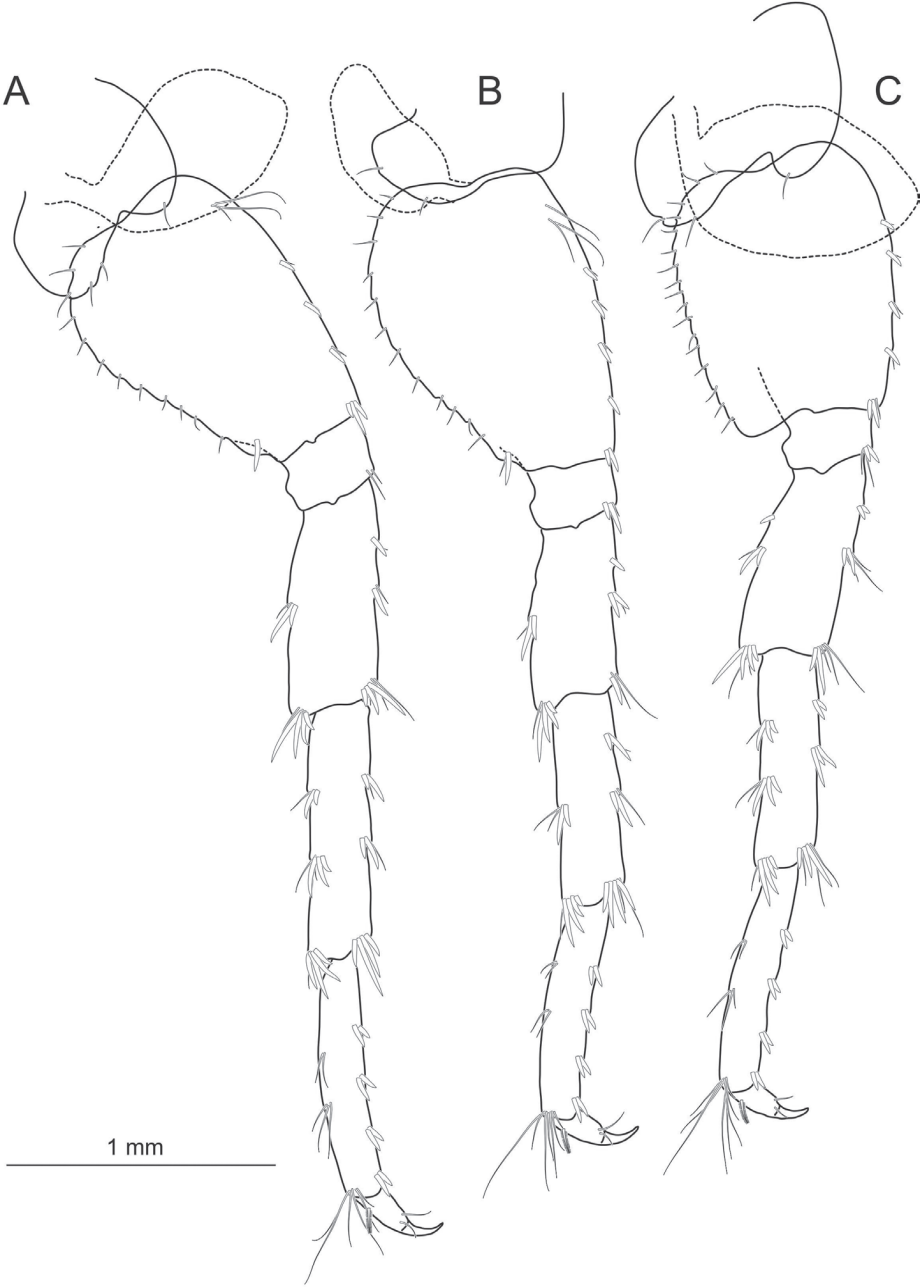
*Echinogammarusozbeki* sp. nov. holotype male **A** pereopod 7 **B** pereopod 6 **C** pereopod 5. Scale bars: 1 mm.

**Figure 6.**
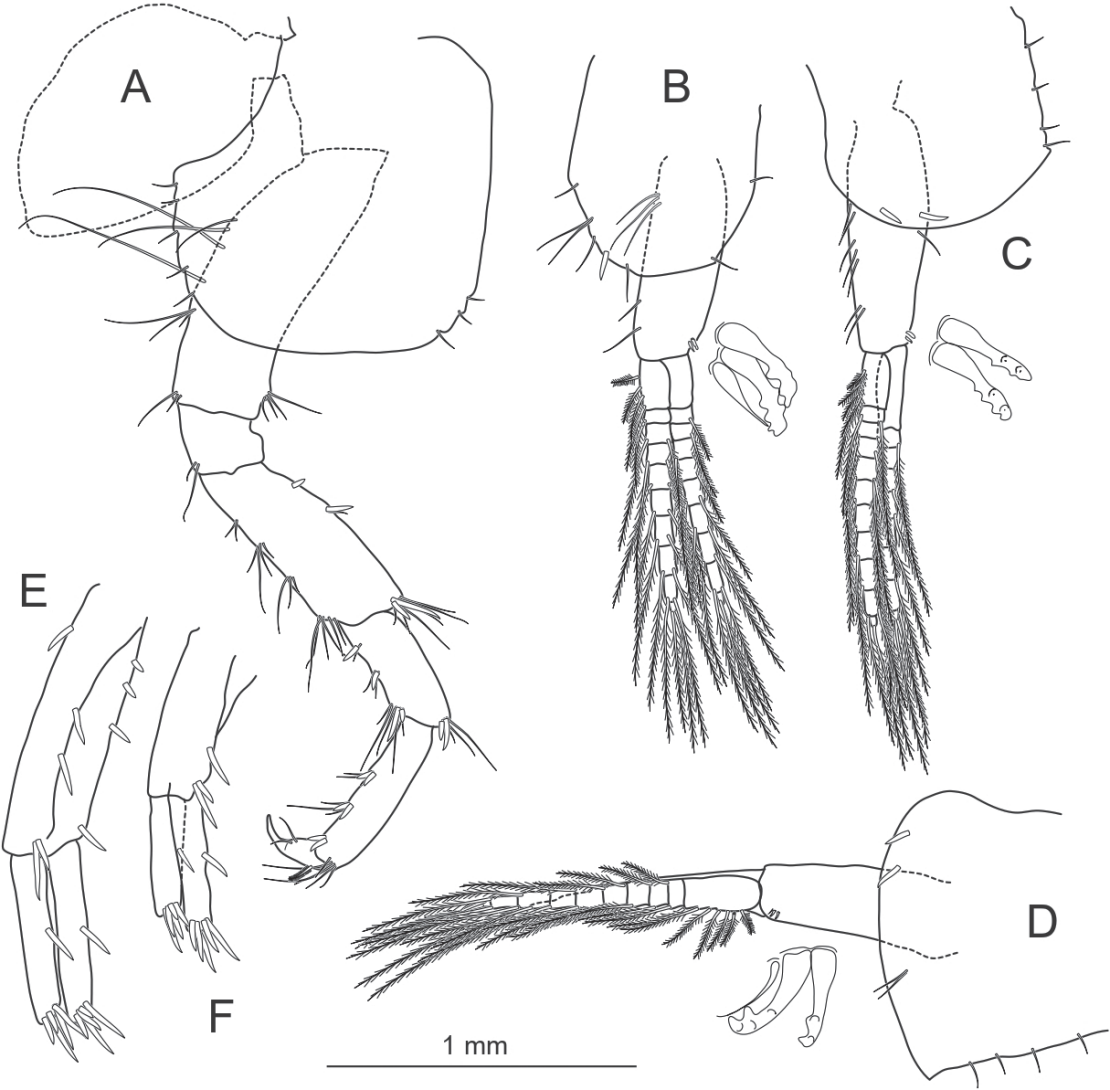
*Echinogammarusozbeki* sp. nov. holotype male **A** pereopod 4 **B** pleopod 1 **C** pleopod 2 **D** pleopod 3 **E** uropod 1 **F** uropod 2. Scale bars: 1 mm.

Figs 2 , 3 , 4 , 5 , 6

#### Type materials.

***Holotype*.** Male, maximum 8.1 mm; Gümüşhane Province, Türkiye (40.081°N, 39.041°E), 17.ix.2019, FFR20000. ***Paratypes*.** 21 males and 13 females, FFR20001, same data as holotype.

#### Etymology.

The new species is named in honour of Prof. Dr Murat Özbek (Ege University, Faculty of Fisheries, Bornova, İzmir). The name is a noun in the genitive singular.

#### Diagnosis.

A small species with kidney-shaped eyes, peduncle segments of antenna 1 bearing a group of long setae distal end, setation of antenna 2 moderate on both peduncular and flagellar segments, urosomites flat, uropod 3 parviramous, inner ramus with 1 or 2 distal spines accompanied by 1 or 2 setae; endopod/exopod ratio 0.21; telson with 1 or 2 setae on outer margin and 1 or 2 setae on the inner surface of each lobe. Anterior and posterior margins of pereopods 5–7 have spines and a few setae.

#### Description.

**Holotype male. Head** (Fig. 2): cephalic lobes rounded, inferior antennal sinus deep, eyes kidney-shaped, shorter than the diameter of the first peduncular segment of antenna 1.

***Antenna 1*** (Fig. 3A) longer than half (~0.75) of the body length, setation weak, peduncle segments bear a group of long setae on the distal end; The length of the setae is 1.2 times the diameter of the segment in which they are implanted. The main flagellum with 19 or 20 segments, accessory flagellum 2- or 3-segmented.

***Antenna 2*** (Fig. 3B) shorter than antenna 1 (0.48 of antenna 1), antennal gland cone straight and reaches to distal end of third peduncular segment, setation moderate on both peduncular and flagellar segments; setae along ventral margins of peduncular segments more than twice as long as diameter of segment; setae on dorsal side of peduncular segments shorter; flagellum with 7 or 8 segments, calceoli absent.

***Gnathopod 1*** (Fig. 3D): basis with 5 or 6 long setae on both posterior and anterior margins; (up to 2.0 times longer than diameter of segment); ischium and merus with a single group of setae on posterodistal corner; carpus ~0.68 of propodus; propodus with 1 medial palmar spine and 3 palmar angle spines, and a few setae on inner surface; dactylus long, with 1 seta on outer margin.

***Gnathopod 2*** (Fig. 3E): basis with long setae on anterior and posterior margins (up to 2.0 times longer than diameter of segment); ischium and merus as on gnathopod 1; carpus ~0.82 of propodus; length of propodus ~0.5 of its width; superior lateral setae bear 3 or 4 groups of setae rows, 1–5 setae in each row; inferior margin bears some setae groups. Propodus palm bear 2 strong and 2 short median palmar spines and short simple setae; without spine on inner surface of propodus.

***Pereopod 3*** (Fig. 3G): basis bears 2 groups of long setae and 2 setae in each group on posterior margin; 4 or 5 setae on anterior margin; ischium bears 3 or 4 posterodistal seta; merus bears 3 rows of setae and 3 or 6 setae in each row on posterior margin and a setal group at posterodistal tip; 2 rows of spines and setae (order of setas and spins: 1 spine + 1 seta) on anterior margin; 1 strong and 1 small spine and 1 group of long setae in the anterodistal tip; setae longer than diameter of segment. Carpus bears 3 groups of spines and setae on posterior margin (order of setas and spins: 2 spines + 3 setae, 2 spines + 3 or 4 setae, 1 strong spine + 3 setae); bald anterior margin; 1 strong spine and 2 long setae in the anterodistal tip; propodus bear 3 groups of spines and setae on the posterior margin, order of setas and spins 2 spines + 1 seta; bald anterior margin; 3 or 4 setae in the anterodistal tip; 1 plumose seta on dorsal margin; dactylus with 2 simple setae at hinge of nail.

***Mandible*** (Fig. 4G, H): incisor with 4 teeth; lacinia mobilis with 4 dentitions; molar triturative; first article of palp without setae, second article bears 4 or 6 distal setae, third article armed with three group E setae, 16 group D setae, and 2 group A setae.

***Upper lip*** (Fig. 4E) with minute setae in distal part.

***Lower lip*** (Fig. 4D): inner lobe absent, apical margin with minute setae.

***Maxilla*** (Fig. 4B, C): inner plate of maxilla 1 with ~11 marginal setae, outer plate with 8 serrated spine teeth; article 2 of right palp with 4 blunt spines, article 2 of left palp with 6 slender spines.

***Maxilla 2*** (Fig. 4F): inner plate with 7 simple facial setae, implanted subparallel to the inner margin; 6 apical setae on inner plate, 7 apical setae on outer plate.

***Maxilliped*** (Fig. 4A): inner plate with 1 subapical spine, 3 blunt apical spine, 5 or 6 marginal setae; outer plate 8 marginal setae and 7 or 8 apical setae.

***Pereopod 4*** (Fig. 6A) shorter than P3; basis bear 4 or 5 long setae on the posterior margin, 1 long seta on anterior margin; 2 setae on anterodistal and posterodistal tip; ischium bears 2 posterodistal setae; merus with 4 groups of setae on posterior margin, 2 spines and 1 seta on anterior margin, 1 strong and 1 small spines and 3 or 4 setae on anterodistal tip; carpus short, with 3 groups of spines and setae on posterior margin (order of setas and spins: 1 spine + 1 seta); bald anterior margin; propodus bear 3 groups of spines and setae on posterior margin; bald anterior margin, 4 or 5 setae in anterodistal; 1 plumose seta on dorsal margin, dactylus with 2 simple setae at hinge of nail.

***Pereopod 5*** (Fig. 5C): basis with a row of 4 spines on anterior margin; posterior margin of basis slightly convex, serrated, with sequential setae; ischium with 2 spines and 2 setae on anterodistal tip, merus with 3 groups of setae and spines on anterior margin, 2 groups of spines and setae on posterior margin; carpus with 3 groups of spines on anterior tip, 3 groups of spines on posterior margin; propodus with 3 groups of spines on anterior margin, 2 groups of setae on posterior margin; dactylus with 2 simple seta at hinge of nail.

***Pereopod 6*** (Fig. 5B): basis convex, without setae on inner surface, 1 spine at posterodistal portion, with sequential setae posterior margin; with 4 sequential spines on anterior margin; ischium with 2 spines and 1 seta on anterodistal tip; carpus with 3 groups of spine + seta combination on anterior margin, 2 groups of spine + seta combination on posterior margin; propodus with 3 groups of spines on anterior margin, 2 groups of setae on posterior margin, 1 plumose seta on dorsal margin; dactylus with 2 simple setae at hinge of nail.

***Pereopod 7*** (Fig. 5A): basis with a row of 4 spines on anterior margin; serrated; without setae on inner surface; with sequential setae on posterior margin; 1 anterodistal spine; anterodistal corner of ischium with some setae; merus shorter than carpus, 2 groups of small spines on anterior margin, 1 group of spines on posterior margin; some spines and setae on anterodistal and posterodistal corners; carpus 2 with group of small spines on both anterior and posterior margins, some spines and setae on anterodistal and posterodistal corners; propodus same as propodus of pereopod 6, 1 plumose seta on dorsal margin; dactylus with 2 simple seta at hinge of nail.

***Uropod 1*** (Fig. 6E): outer ramus 0.67 and inner ramus 0.68 of length of peduncle, respectively; peduncle bearing 1 proximal spine, 2 inner marginal spines, 2 outer marginal spines; 2 strong ramal spines; 1 inner ramus spine; 1 outer ramus spine; 3 or 4 strong terminal spines.

***Uropod 2*** (Fig. 6F): outer ramus 0.83 and inner ramus 0.81 of length of peduncle, respectively; peduncle bearing 1 proximal spine, 1 inner marginal spine, 1 outer marginal spine; 1 strong ramal spine; 1 inner ramus spine; 1 outer ramus spine; 3 or 4 strong terminal spines.

***Uropod 3*** (Fig. 3F): parviramous, inner ramus with 1 or 2 distal spines accompanied by 1 or 2 setae; peduncle with 2 strong paired spines; endopod/exopod ratio is ~0.21; article 1 of outer ramus with 2 groups of spines and setae on both outer and inner margins (order of setas and spins: 1 spine + 3 or 4 setae); 2 or 3 distal spines and long setae on the exopod; terminal segment short.

***Urosomites***: all urosomites not elevated; urosome 1 without setae or spines; urosomes 2 and 3 bear 1 seta and 1 spine each.

***Telson*** (Fig. 3C): cleft, each lobe with 1 strong distal spine and 2 or 3 long setae; 1 or 2 setae on outer margin and 1 or 2 setae on inner surface of each lobe.

***Coxal plate 1*** (Fig. 3D) rectangular, 2 anterodistal setae and 1 posterodistal seta; lower margin with 4–7 setae; glabrous inner surface.

***Coxal plate 2*** (Fig. 3E) rectangular, 1 anterodistal seta and 1 posterodistal seta; lower margin with 2 or 3 setae; glabrous inner surface.

***Coxal plate 3*** (Fig. 3G) rectangular; 2 anterodistal setae and 1 posterodistal seta; lower margin with sequential setae; glabrous inner surface.

***Coxal plate 4*** (Fig. 6A) excavated, almost equal in width and length; lower margin with sequential setae; 4 setae in the posterior margin; 2 or 3 setae on anterodistal corner; glabrous inner surface.

***Coxal plate 5*** (Fig. 5C): anterior lobe bearing 1 seta and 3 setae on the ventral margin of posterior lobe.

***Coxal plate 6*** (Fig. 5B): posterior lobe bearing 1 seta on ventral margin.

***Coxal plate 7*** (Fig. 5A): posterior lobe bearing 3 setae on ventral margin.

***Epimeral plate 1*** (Fig. 6B) rounded; 1 spine on anterodistal margin, 1 spine on posterodistal margin.

***Epimeral plate 2*** (Fig. 6C) rounded, without point; with two spines on ventral margin, 3 sequential setae on posterior margin.

***Epimeral plate 3*** (Fig. 6D) with 2 spines on ventral margin; 2 or 3 setae on posterior margin, without point posterodistally.

***Pleopods 1–3*** (Fig. 6B–D) subequal; peduncle with some setae and 1 or 2 retinacula; rami with 10 or 11 segments and numerous plumose setae.

**Female (Fig. 2).** Body 6.8 mm long.

***Antenna 1*** less setose than that of male, first peduncle segments with long setae on distal portion, with 17–21 segments on flagellum, 2 segments on accessory flagellum. Gnathopod 1 and 2 similar to that of male, setation more intense on propodus, dactyli of gnathopods 1 and 2 long. Uropod 3 similar to that of male, distal part of peduncle with spines; inner and outer margins of outer lobe more densely setose than male outer lobe with row of 2 spine and 5 or 6 setae in each margin. Telson similar to that of male, each lobe with 1 or 2 distal spines accompanied by 1 or 2 setae longer than the spines.

Oostegites present on gnathopod 2 through to pereopod 5.

#### Variability.

The number of flagellar segments of Antenna 1 was determined as 16 in six paratype individuals, while Antenna 2 had nine flagellar segments. The number of setae on the inner and outer edges of the exopod varied between three and six.

## ﻿Discussion

In this study, *Echinogammarusozbeki* sp. nov. is compared with species from the family Gammaridae, and their similarities and differences are emphasized. At first glance, *E.ozbeki* sp. nov. closely resembles *Gammaruspageti* Mateus & Mateus, 1990 and *Gammarusbalcanicus* Schäferna, 1923. *Gammarusbalcanicus* is known for its wide distribution range and intraspecific variation. Karaman and Pinkster, (1987) noted that the endopod (uropod 3) of *G.balcanicus* collected from Keklik (Erzurum province) is relatively short. *Echinogammarusozbeki* sp. nov. is similar to *G.balcanicus* in having spines on the pereopods, relatively little setation on all appendages, and a short endopod. However, it differs from *G.balcanicus* in the length/width ratio of the telson lobes, the presence of long setae on the peduncular segments of antenna 1, moderate setation on all extremities, long setae on antenna 2, and setae and spines on the outer and inner margin of the exopod 3. Mateus and Mateus (1990) emphasized the short endopod of *G.pageti* collected from near Maden (Erzurum province). *Echinogammarusozbeki* sp. nov. is similar to *G.pageti* in its exopod/endopod ratio, but differs from by having a parviramous type uropod 3, a row of setae and spines on the gnathopods, and the setation of the mandibular palp.

*Echinogammarusozbeki* sp. nov. shows similarities in general body appearance to individuals belonging to the genus *Turcogammarus*. *Turcogammarusaralensis* (Uljanin, 1875) and *T.spandli* (Karaman, 1931) have been recorded from nearby river basins, and one species, *T.turcarum* (Stock, 1974), has been recorded from Anatolia. *E.ozbeki* sp. nov. differs from *T.turcarum* from Mount Ağrı on the Türkiye–Iran border by the absence of long setae on pereopod 7, the presence of short and few setae on the epimeral plate, and the sparser setation of the telson (Karaman 2021). *Litorogammarusdursi* Marin, Palatov & Copilaș-Ciocianu, 2023, described from the southwestern Caucasus region and the Durso River, and belonging to the Pontocaspian region, exhibits similar characteristics to the new species, such as the exopod/endopod ratio, the absence of carina on metasome segments, and the presence of sequential spines and setae on the exopod of uropod 3 (Marin et al. 2023). However, *E.ozbeki* sp. nov. differs from *L.dursi* in having setae on the inner surface of coxal plate 3 absent, less dense setal rows on the mandible and lower lip, curled setae on antenna 2 absent, and a 2- or 3-segmented accessory flagellum. Recent molecular data and studies have confirmed that *Chaetogammarustrichiatus* and *Trichogammarustrichiatus* are synonyms (Copilaș-Ciocianu et al. 2023). *Chaetogammarustrichiatus* (= *Echinogammarustrichiatus*) is known to occur in Türkiye (İpek and Özbek 2022). The morphological differences between *E.ozbeki* sp. nov. and *E.trichiatus* species are compared in the Table 1.

**Table 1.** Comparison of some distinct characters of *Echinogammarus özbeki* sp. nov. with species of the genus *Echinogammarus* distributed in Türkiye.

***Echinogammarusozbeki* sp. nov.**: a small species; urosome segments without elevation; inner surface of basis of pereopods 5–7 without setae; all extremities without curled setae; all extremities have sparse setae	***Echinogammarusantalyae* G. Karaman, 1971**	Urosome segments elevated; exopod of uropod 3 with densely plumose setae; basis of pereopods 5–7 with some setal groups on inner surface
	***Echinogammarusbaliki* Özbek & Ustaoğlu, 2007**	Exopod of uropod 3 with plumose setae; urosomite 3 dorsally elevated; outer margin of telson lobes with spines and setae; accessory flagellum with 5 segments; inner surface of gnathopods 1 and 2 with groups of straight and curled setae
	***Echinogammarusfoxi* (Schellenberg, 1928)**	Basis of pereopods 5–7 with some setal groups on inner surface; antenna 2 with weak setation, short setae; flagellum of antenna 1 with 25 segments
	***Echinogammarusischnus* Stebbing, 1899**	Antenna 1, antenna 2, pereopod 3, gnathopods with densely curled setose; telson very short, with 2 lateral spines and 3 dorsal posterior spines; setation absent or very sparse on peraeopods 5–7.
	***Chaetogammarustrichiatus* (*Echinogammarustrichiatus*) Martynov, 1932**	Antenna 2, uropod 3 and gnathopods with dense, curled setae
	***Echinogammarusstocki* G. Karaman, 1970**	Antenna 1 with relatively long setae; telson lobes longer than wide; exopod of uropod 3 have dense, long, simple setae
	***Echinogammarusveneris* (Heller, 1865)**	Antenna 2, exopod of uropod 3 and pereopod 3 have dense, simple setae; basis of pereopod 5–7 have some seta groups on inner surface; urosome segments slightly elevated

The genus *Echinogammarus* has been divided into three main groups—*E.berilloni* group, *E.pungens* group, and *E.simoni* group—according to morphological features. *Echinogammarusozbeki* sp. nov. differs from the *E.berilloni* group by the absence of dense setae. Species of the *E.berilloni* group have a long, dense setae on the different extremites.

*Echinogammarusberilloni* has dense setae on the metasome and urosome segments. *Echinogammaruscalvus* has long curled setae on the gnathopods. *Echinogammarusmeridionalis* possesses long, straight, and curled setae on antenna 2 and gnathopods. *Echinogammarusaquilifer* has dense setae only on the flagellum of antenna 2. On the other hand, *E.zebrinus* bears spines and setae on the urosome and metasome segments. *Echinogammarusozbeki* sp. nov. is distinct from other members of the group due to the absence of dense setae or spines on its similar appendages, especially on the metasome segments and the inner surface of the bases of the pereiopods. The shape and setal arrangement of the propodus and weak elevation of the urosome segments in *E.cari*, and the inflated structure of the peduncle segments of antenna 2, as well as the dorsal elevation of the urosome segments in *E.tibaldii* (Karaman, 1973) facilitate the differentiation of *E.ozbeki* sp. nov. from these species. *Echinogammarusozbeki* sp. nov. is distinguished from the *E.pungens* group by the absence of setae on the inner surface of the pereopod basis, the lack of elevation on the urosome segments, the shape of the epimeral plates, and the rare occurrence of spine-setae (Pinkster 1973).

The morphological features of the *E.ozbeki* sp. nov., which differs from the species belonging to the genus identified so far in Türkiye, are provided in Table 1.

## Supplementary Material

XML Treatment for Echinogammarus ozbeki

## ﻿Additional information

### Author ORCIDs

Hazel Baytaşoğlu https://orcid.org/0000-0002-9959-1829

### Data availability

All of the data that support the findings of this study are available in the main text.
